# Shifting the Paradigm on Cannabis Safety

**DOI:** 10.1089/can.2020.0003

**Published:** 2022-02-10

**Authors:** Pauric Bannigan, James C. Evans, Christine Allen

**Affiliations:** Leslie Dan Faculty of Pharmacy, University of Toronto, Toronto, Canada.

**Keywords:** recreational cannabis use, nonmedical cannabis use, side effects, adverse health effects

## Abstract

The global movement toward legalization of cannabis is resulting in an ever-increasing public perception that cannabis is safe. Cannabis is not the first drug to be available for nonmedical use, nor is it the first to have such an unfounded safety profile. The safety of long-term exposure to phytocannabinoids is misunderstood by, and under reported to, the general public. There is evidence to suggest that long-term use of recreational cannabis may be associated with an increased risk of undesirable side effects. This evidence warrants both appropriate caution from the general public and investment in further research by government and industry sectors that are profiting from the sale of these potent psychoactive agents. There is no doubt that these compounds have medical potential. However, in addition to the medical potential, we must also remain aware of the adverse health effects that are becoming synonymous with recreational cannabis use. This perspective highlights the privileged role that cannabis has as a perceived “safe drug” in society and summarizes some concerning side effects that are becoming associated with regular nonprescribed cannabis use.

## Background

Cannabis is often considered to be one of the earliest plants used for medicinal purposes by humans.^1^ It is also arguably more infamous for its nonmedicinal use. As reported by the United Nations, cannabis is the most grown, transported, and sold controlled substance in the world.^2^ To tackle the sale of illegal cannabis, and to make medical cannabis more accessible to those who need it, several countries have legalized cannabis for medical purposes. Some countries have gone a step further and legalized cannabis for recreational use. For example, in late 2018, the sale of recreational cannabis became legal in Canada.

The Cannabis Act initially allowed for “the cultivation, production and sale of dried cannabis flower, oil tinctures and oil capsules.”^3^ One year postlegalization, the Cannabis Act was amended to allow for the sale of cannabis edibles, topicals, infused beverages, vapes, and concentrates.^3^ Since legalization in Canada, several other countries around the world have made significant strides to follow suit, including Germany, Mexico, and the United States.^4–6^ Cannabis is not the first drug to be legally available for nonmedical use. Tobacco, alcohol, and caffeine have been freely available to the public for recreational use for decades. However, none of these substances are risk free, and all have associated side effects. These vary in severity depending on the substance, frequency of use, and dose administered (Table 1).

**Table 1.** List of Common Substances/Drugs Available for Recreational Use, and their Corresponding Side Effects

Drug/Substance		References
Select side effects associated with overconsumption/long-term use		
Alcohol	• Certain types of cancer	^90^
	• High blood pressure, heart disease, stroke, and liver disease	
	• Mental health problems	
	• Dependency	
Caffeine	• Nausea	^91^
	• Dysphoria	
	• Insomnia	
	• Headache	
Tobacco	• Lung, esophageal, larynx, and oral cavity cancers	^92^
	• Various lung diseases	
	• Coronary heart disease	
	• Gum disease and tooth loss	

While a lethal overdose of cannabis seems unlikely in humans,^7^ this is perhaps not the best barometer to assess the safety of cannabis or its principle psychoactive component, Δ^9^-tetrahydrocannabinol (Δ^9^-THC). Afterall, smoking tobacco is unlikely to lead to a lethal nicotine overdose, yet this does not mean that tobacco is risk free. There is popular misconception regarding the safety of cannabis, and as academics interested in the clinical potential of cannabinoids, we feel duty bound to highlight these issues. Our motivation in writing this article is to stress that just because nonmedical cannabis use is becoming legal in many parts of the world doesn't mean it is safe.

## Educating the Public and Breaking the Stigma

**FIG. 1.**
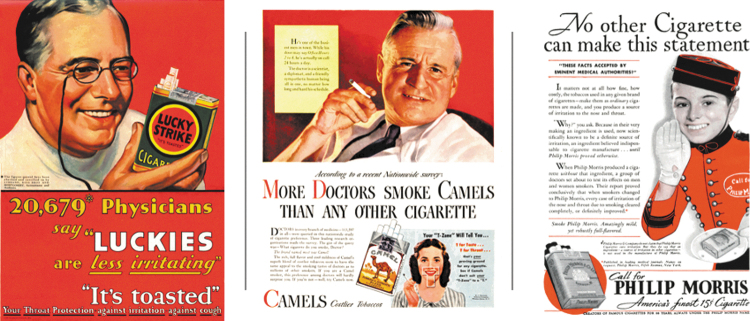
Posters from the early 1900s, taken from the collection of Stanford Research Into the Impact of Tobacco Advertising (tobacco.stanford.edu). Color images are available online.

The safety profiles of recreational drugs are notoriously difficult to establish. It took 60 years for the damaging health effects of tobacco smoking to become mainstream knowledge.^8^ As the link between smoking and lung cancer was being established knowledge mobilization remained a challenge. This is in part due to the addictive nature of cigarette smoking and a consequence of the powerful marketing campaigns run by tobacco companies to influence the public (Fig. 1). In the case of cannabis, we now see extraordinary health and safety claims for this once controlled substance. These claims are usually spread via online forums and through social media, with only so-called “anecdotal evidence” offered for validation. The fact that cannabis is a natural substance and its use is becoming permissible across the globe should not be sufficient for people to think it is harmless. In reality there is very little evidence for many of the safety and medical claims that are made regarding cannabis. As researchers working in this space it is difficult to navigate the current landscape that is often littered with misinformation. In addition to this we must battle the stigma that is associated with researching these compounds. Our interest, along with that of many other researchers entering this space, stems from the potential of these exogenous cannabinoids to modulate the processes influenced by the human endocannabinoid system (ECS). These processes include appetite and energy balance, cardiovascular functions, cell apoptosis, neuronal development, immune modulation, neuronal plasticity, and reproductive functions.^1^

Several exogenous cannabinoids have been shown to bind to human cannabinoid receptor 1 (CB1) and/or human cannabinoid receptor 2 (CB2). These cannabinoid receptors are ubiquitously expressed in almost every part of the human body. CB1 receptors are mainly located within the central nervous system,^9^ while CB2 receptors are found primarily on cells of the hematopoietic and immune systems.^10–12^ Interactions between certain exogenous cannabinoids and the human ECS also result in the sought-after recreational effects of cannabis. Despite the potential for abuse, there is growing evidence to support the clinical application of exogenous cannabinoids in certain disease states. This is exemplified by the FDAs recent approval of the plant-derived cannabidiol (CBD) formulation, Epidiolex^®^ (GW Pharmaceuticals). Epidiolex is currently approved in the United States and Europe for the treatment of two rare forms of childhood epilepsy (Lennox-Gastaut Syndrome and Dravet Syndrome).

Even with the advances that have been made in the science of cannabinoids, there is still much we do not know about these compounds. Of concern, is the lack of safety data available for long-term exposure to these compounds (particularly in children and adolescents).

## Cannabis Use and Mental Health

The question of how cannabis use affects mental health is of significant interest to both researchers and society. The harmful effects related to cannabis use may be amplified by both the degree of exposure and the age of initiation.^13^ As such, there is considerable concern that cannabis use among adolescents, young adults, and vulnerable individuals, may contribute to the development of mental health conditions, including anxiety and depressive disorders, use disorder, and psychosis.^14^

For younger adults and adolescents, it is postulated that this increased risk may be due to the interference of phytocannabinoids, particularly Δ^9^-THC, with ongoing brain development.^15^ Conversely, 16–24 year olds may be found to be most at risk because they comprise the portion of the population most likely to consume cannabis,^2,16^ and therefore the population most likely to experience the associated side effects.^13,17–19^ In addition, some vulnerable individuals may be more susceptible to developing various mental health disorders, regardless of cannabis consumption, but rather due to certain biological and sociological factors.^20^

Despite these confounding factors, there is growing evidence to suggest that cannabis use may have an impact on normal brain function and consequently may have a negative impact on mental health.

### Cognitive impairment

Δ^9^-THC activates CB1 receptors in the brain to elicit its intoxicating effects. These effects tend to vary between individuals for several reasons including differential rates of Δ^9^-THC absorption and different levels of tolerance to Δ^9^-THC. Although not itself lethal, the impaired motor and cognitive function arising due to cannabis intoxication (i.e., Δ^9^-THC intoxication) is often associated with poor decision making and an increased risk of personal injury.

There is experimental evidence, from driving simulators, to suggest that cannabis intoxication impairs several cognitive skills related to driving ability.^21^ Despite this experimental evidence, the findings from epidemiological studies are not as clear, largely due to confounding factors such as multiple substance use, in particular concurrent consumption of cannabis and alcohol.^21–23^

However, overall the effects of cannabis intoxication have been linked with an increased risk of motor vehicle crash.^24^ Some studies conducted in the United States and Europe have suggested that crash risk may increase twofold to threefold due to cannabis intoxication.^21,23,25^ Although significant, the effects of alcohol intoxication are more substantial, and estimated to increase crash risk between 6- and 15-fold.^25^ Unsurprisingly, concurrent consumption of both cannabis and alcohol increases the risk of car accidents substantially more than consumption of either substance alone.^21,23–25^

### Use disorder/addiction

Regular cannabis use can develop into an addiction in much the same way that addiction develops to other substances, such as opioids or tobacco. For example, the typical “high” felt by users yields the desire for repeated use, and for some cannabis users this desire can develop into cannabis use disorder (CUD).^26^ Cannabis users with earlier initiation times, that is, who begin their cannabis use earlier in life, and cannabis users with more exposure (i.e., more heavy cannabis use), are widely considered more at risk of developing CUD.

Some epidemiological studies have concluded that CUD has been one of the most common addictions, after alcohol and tobacco, over the past 20 years in Australia, Canada, and the United States.^25^ It is estimated that CUD affects 1–2% of adults who have used cannabis in the past year and 4–8% of adults who have used cannabis in their lifetimes.^25,27,28^ Overall, compared to other drugs of misuse (nicotine [32%]; heroin [23%]; cocaine [17%]; alcohol [15%]; and stimulants [11%]^25,29,30^), the rate of dependence on cannabis is relatively low (9–10%).^25,28,31^ Additionally, there is evidence that the “adverse health and social consequences associated with cannabis dependence are less severe than those associated with alcohol or opioids.”^32^

However, this does not mean that CUD should be regarded as a trivial illness. The majority of individuals who suffer from CUD seek professional attention to overcome this addiction. In the past two decades there has been a steady increase in the number of cannabis users seeking assistance to control or quit cannabis use in Europe, Australia, and the United States.^25^ In North American jurisdictions that have implemented new cannabis laws, there is potential for CUD rates to increase in the near future due to increased availability of cannabis. It has been estimated that there are almost 1 million and 10 million daily or near-daily users of cannabis in Canada and the United States, respectively.^2^ A recent study that tracked the rates of CUD following recreational legalization of cannabis in certain U.S. states, from 2008 to 2016, showed an increase in the risk of CUD in 12–17 year olds, and increased rates of CUD in adults over 26 years of age.^32^

The effects of cannabis legalization on rates of CUD in Canada have not yet been reported.

### Psychosis

Several studies across different ethnic groups have shown that cannabis users with younger initiation times are more likely to have psychotic symptoms during adulthood.^13,20,33–36^ A recent multicenter case–control study suggests that daily high potency cannabis use (≥10% Δ^9^-THC) can result in an almost fivefold increase in the likelihood of being diagnosed with psychosis, compared with individuals who have never used cannabis.^17^ While other studies, that looked primarily at populations from the United States, Australia, New Zealand, and Europe, have suggested a two and four fold increase in risk of developing psychosis, for regular cannabis and heavy cannabis users, respectively.^20,37,38^ However, despite the findings from these studies, there is currently insufficient evidence to support an association between cannabis use and psychosis. Most psychosis patients have never used cannabis, and many cannabis users do not develop psychosis.^25,34,39^

In summary, initiation time and regular cannabis use may be connected to a higher risk of psychotic episodes; however, current evidence implies that cannabis use is just one factor in a complex collection of factors that can contribute to the development of psychosis.^34,39^ Other risk factors include age, in addition to, genetic and sociological factors.^13,17–20^

### Anxiety disorders

In a 2017 survey of Canadians, over 50% of respondents declared that cannabis use improved their anxiety.^40^ However, current research does not align well with these survey results.

Data from the “Netherlands Mental Health Survey and Incidence Study” and the “National Epidemiological Survey on Alcohol and Related Conditions” (NESARC) in the United States identified cannabis as a potential causal factor in developing anxiety disorders, or in exacerbating symptoms in people who suffer from anxiety disorders.^41–44^ Other studies, confined to residents of Miami and Seattle, reported correlations with only certain types of anxiety disorders.^45,46^ While, studies on populations from Australia, the United Kingdom, Sweden, and the United States, wherein confounding factors such as childhood experience and cigarette use were controlled for, found no such associations.^47–51^ Preclinical and clinical research has suggested that the duration and dose of Δ^9^-THC administered may be correlated with anxiolytic and/or anxiogenic responses.^52–54^

Overall, more research is necessary to investigate the potential relationships between cannabis use and anxiety disorders.^20^

### Depressive disorders

Similarly, some studies suggest that cannabis use can be correlated with the likelihood of being diagnosed with a depressive disorder. One long-term cohort study, namely, the “Cambridge Study in Delinquent Development,” reported that cannabis use, in particular with early initiation, increased the risk of being diagnosed with depression.^55^ Studies conducted on Australians, Swiss, and Canadians (Nova Scotians) reported this correlation to be more prominent in chronic cannabis users^56,57^; yet, with weekly cannabis use being enough to cause a depressive disorder.^56,58^ However, as is the case with anxiety, when controlling for confounding factors such as use of other substances, early childhood experience, and education, other studies conducted on Australians, Danes, and Americans (NESARC) reported no clear association between depression and cannabis use.^47,59,60^

## Cannabis Use and Physical Health

Cannabinoids have been linked to a plethora of other side effects related to physical health. The majority of these are not yet well established and require further research. For example, the recent clinical approval of Epidiolex by the U.S. FDA included a request for nine additional clinical trials to better understand the impact of long-term CBD exposure on healthy volunteers.^61^ Given the extent to which the ECS is thought to regulate homeostasis, the side effects associated with phytocannabinoids, and in particular Δ^9^-THC, may be quite far reaching.

### Vascular health

A recent review by Pacher et al., summarizes the effects of cannabis on cardiovascular health.^62^ Δ^9^-THC is a partial agonist of the CB1 receptor. The cardiovascular effects of recreational cannabis use can vary depending on Δ^9^-THC content of the product and the route of administration, that is, the effects of Δ^9^-THC are believed to be dose dependent.^62^ High doses of Δ^9^-THC have been shown to exert CB1 receptor mediated proinflammatory, pro-oxidant, and profibrotic effects. At low doses, Δ^9^-THC may exert CB2 receptor mediated tissue protective and anti-inflammatory effects.^62^ In rare cases acute exposure to Δ^9^-THC can result in life-threatening cardiovascular effects, such as myocardial infarction and ischemia.^62^ Some studies suggest these risks are higher in older adults.^25^ However, there have been numerous reports of adverse cardiovascular events in healthy young cannabis smokers.^63–66^ While more research is needed to fully determine the effect of cannabis and Δ^9^-THC on cardiovascular health, the preliminary evidence certainly warrants caution.

### Gastrointestinal health

The ECS functions in the brain and the GI tract to help regulate energy balance and food intake. Δ^9^-THC can increase food uptake and inhibit gastric motor activity via CB1 receptor activation.^67,68^ Homeostasis in the gastrointestinal tract can be interrupted by chronic cannabis use. For example, cannabinoid hyperemesis syndrome is a rare condition that involves cyclic nausea and vomiting, which can be induced by long-term and intense cannabis use.^67^ The discovery that cannabinoid receptor agonists can influence gastrointestinal motility has made the ECS a possible target for the treatment of some gastrointestinal diseases such as Crohn's disease and ulcerative colitis. However, a major limitation for such cannabinoid-based gastrointestinal therapies is their potential side effects.^67^

### Cancer risk

Given the established link between tobacco smoking and oncogenesis,^69^ it is reasonable to assume that comparable consequences may be associated with cannabis smoking, as both involve the burning and inhalation of dried plant material. Several studies conducted in North African, North American, European and Australian populations examined a possible link between cannabis smoking and oncogenesis.^70–75^

These data are summarized in a recent systematic review and meta-analysis by Ghasemiesfe et al.^71^ Briefly, for the majority of cancer types investigated, there is currently no strong evidence to support any association between cannabis smoking and oncogenesis.^71^ However, there is emerging evidence to suggest that daily use of cannabis is associated with an increased risk of developing testicular germ cell tumors.^71^ Three separate case–control studies suggest that the resulting increased risk could be up to twofold.^76–78^

In summary, long-term studies of cannabis only smokers are necessary to improve our understanding of any possible associations that might exist between cannabis use and certain types of cancer, in particular lung and oral cancers, which are commonly associated with tobacco smoking.^71^

### Reproductive function

There is limited *in vivo* evidence to suggest that cannabis can negatively affect testosterone production and sperm mobility in men.^79,80^ In rats, the administration of “high levels of Δ^9^-THC has been shown to inhibit ovulation.”^81,82^ A recently published study has suggested that long-term exposure to Δ^9^-THC may result in infertility in men.^83^ Despite these potential links between cannabis use and impaired fertility, we currently lack concrete clinical evidence to understand the degree of risk. However, it is likely advisable for both men and women to avoid cannabis use when trying to conceive.

## Outlook

In the past few decades the amount of Δ^9^-THC present in cannabis plants has been steadily increasing. In samples confiscated by the U.S. DEA the average potency of Δ^9^-THC has increased more than fourfold, from 4% (1995) to 17% (2017).^84,85^ This trend is not confined to the United States, nor to dried cannabis flower. Data from the United Nations show that black market cannabis concentrates and edibles typically contain up to 69% Δ^9^-THC, and in recent years there has been a five fold increase in the number of such products with greater than 75% Δ^9^-THC content.^2^

In Canada, licenced dispensaries are now stocking dried cannabis flowers that contain up to 30% Δ^9^-THC; High Tide Kade's Kush is available on the Alberta Cannabis website with a reported potency of 17–30% Δ^9^-THC.^86^ The Canadian government's recent approval of cannabis concentrates, cannabis edibles, cannabis beverages, and cannabis vape devices, is likely to lead to more offerings of high Δ^9^-THC potency products in 2020. This increase in availability of high Δ^9^-THC cannabis products in North America, along with the large numbers of daily or near-daily users,^2,32^ may exacerbate the number, type, and severity of cannabis-related side effects reported in the coming years.

One of the most common myths associated with cannabis is that it must be safe because it is a natural product and has been used for thousands of years. This misconception is reinforced through internet forums and social media, where cannabis is often described as a “panacea” for a wide range of medical conditions with only “anecdotal evidence” provided for support. The astrophysicist Carl Sagan once said that “extraordinary claims require extraordinary evidence.” In the current climate of increasing cannabis legality there appears to be no end to the “extraordinary claims” that people are willing to make regarding the safety and efficacy of this plant. However, we are nowhere near the “extraordinary evidence” necessary to back these claims, and while the evidence for the cannabis “panacea” may be lacking, the evidence for side effects associated with cannabis use is certainly not. 

Contradicting these widespread health claims and educating the public on the risks of cannabis use are not easy tasks. Academic researchers and health care professionals have called for more to be done to change the public's attitude toward cannabis. As a scientific community we need to significantly increase our understanding of both the potential benefits and harms associated with acute and long-term exposure to cannabis and cannabinoids.

There is a fear that today's misconceptions regarding the safety of cannabis use may be judged by future generations in the same way that our generation now critiques the tobacco smoking era.

These fears extend beyond Δ^9^-THC. Perhaps one of the most concerning trends has been the recent use of cannabinoid products during pregnancy. Often these are CBD oils that are used as a “natural” remedy for nausea experienced during pregnancy.^87^ CBD and other cannabinoids have been shown to cross the blood-placenta barrier.^88^ Children who are prenatally exposed to cannabinoids are more likely to experience several developmental disorders, such as inattention, lower IQ scores, and academic underachievement.^31^ A recent preclinical study reported that cannabinoid exposure via lactation, “can delay a milestone of early childhood development, the trajectory of GABAs polarity transition,” which can impact early-life communication.^89^

**FIG. 2.**
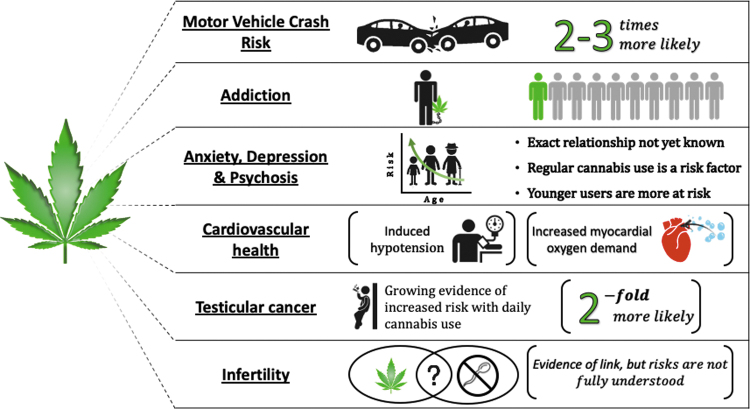
Diagram depicting some of the significant consequences and adverse effects that have been associated with recreational cannabis use. Color images are available online.

In summary, there is a growing body of evidence to suggest that regular cannabis use may have long-term health implications; these may include physical dependency and/or addiction, cognitive impairment, psychosis, anxiety and depressive disorders, cardiovascular issues, infertility, and possibly an increased risk of certain types of cancer (Fig. 2). However, even when the scientific literature on the health implications of cannabis use is examined (rather than internet forums, blogs, news headlines, etc.) contradictory data are found. It is clear that significantly more research is needed to fully understand the impact that recreational cannabis use has on our mental and physical health. The cannabis companies and governing authorities who are profiting from the legalization and sale of cannabis products to the general public should reinvest a portion of these funds into long-term safety studies of these compounds.

## Abbreviations Used

Δ^9^-THC: Δ^9^-tetrahydrocannabinol
CB1: cannabinoid receptor 1
CB2: cannabinoid receptor 2
CBD: cannabidiol
CUD: cannabis use disorder
ECS: endocannabinoid system
